# Rigidity with Flexibility: Porous Triptycene Networks for Enhancing Methane Storage

**DOI:** 10.3390/polym16010156

**Published:** 2024-01-04

**Authors:** Fei Guo, Hui Ma, Bin-Bin Yang, Zhen Wang, Xiang-Gao Meng, Jian-Hua Bu, Chun Zhang

**Affiliations:** 1 National Engineering Laboratory for Advanced Yarn and Fabric Formation and Clean Production, Technology Institute, Wuhan Textile University, Wuhan 430200, China; guofeifei0806@163.com; 2 College of Life Science and Technology, National Engineering Research Center for Nanomedicine, Huazhong University of Science and Technology, Wuhan 430074, China; dongji0828@126.com (H.M.); 17371266359@163.com (B.-B.Y.); chunzhang@hust.edu.cn (C.Z.); 3 School of Chemistry, Central China Normal University, Wuhan 430079, China; 4 Xi’an Modern Chemistry Research Institute, Xi’an 710065, China; bujianhua@163.com

**Keywords:** triptycene, hypercrosslinked polymers, flexibility, methane storage

## Abstract

In the pursuit of advancing materials for methane storage, a critical consideration arises given the prominence of natural gas (NG) as a clean transportation fuel, which holds substantial potential for alleviating the strain on both energy resources and the environment in the forthcoming decade. In this context, a novel approach is undertaken, employing the rigid triptycene as a foundational building block. This strategy is coupled with the incorporation of dichloromethane and 1,3-dichloropropane, serving as rigid and flexible linkers, respectively. This combination not only enables cost-effective fabrication but also expedites the creation of two distinct triptycene-based hypercrosslinked polymers (HCPs), identified as PTN-70 and PTN-71. Surprisingly, despite PTN-71 manifesting an inferior Brunauer–Emmett–Teller (BET) surface area when compared to the rigidly linked PTN-70, it showcases remarkably enhanced methane adsorption capabilities, particularly under high-pressure conditions. At a temperature of 275 K and a pressure of 95 bars, PTN-71 demonstrates an impressive methane adsorption capacity of 329 cm^3^ g^−1^. This exceptional performance is attributed to the unique flexible network structure of PTN-71, which exhibits a pronounced swelling response when subjected to elevated pressure conditions, thus elucidating its superior methane adsorption characteristics. The development of these advanced materials not only signifies a significant stride in the realm of methane storage but also underscores the importance of tailoring the structural attributes of hypercrosslinked polymers for optimized gas adsorption performance.

## 1. Introduction

According to the European Union Commission report stated in 2004, transportation was one of the main sectors contributing to energy consumption and depletion, of which harmful emissions resulted in global warming, climatic deterioration, and air pollution. As a response to these environmental challenges, there has been a growing interest in using natural gas, primarily composed of methane (>95%), as a cleaner alternative to conventional energy sources like oil-derived products [1,2,3]. However, a critical hurdle in realizing the potential of natural gas (NG) as a clean fuel lies in the efficient storage of methane, particularly for applications in transportation [4]. This challenge has spurred research into porous adsorbents with high surface areas and proper pore volumes, which have the potential to enable efficient methane storage. Among these porous materials are molecular cages [5,6], porous organic polymers (POPs) [7,8,9,10], and metal-organic frameworks (MOFs) [11,12,13,14]. Among them, hypercrosslinked polymers (HCPs), formed by a simpler and scalable procedure with irreversible condensation of Friedel–Crafts alkylation or Scholl reaction, possess generally outstanding physicochemical stability compared with MOFs and COFs [15,16,17].

Triptycene and its derivatives, renowned for their captivating three-dimensional and rigid molecular structures, have emerged as focal points of extensive research across diverse domains within chemistry and materials science. Their distinctive structural characteristics, characterized by three fused aromatic rings forming a compact triangular arrangement, bestow upon them a unique and intriguing nature. These compounds are particularly valued for their pronounced steric hindrance, which allows precise control of chemical reactivity, a trait of paramount importance in selective reactions. Furthermore, triptycene’s ability to exhibit chirality makes it invaluable in asymmetric synthesis and as chiral catalysts. Their three-dimensional architecture offers exceptional versatility, permitting the attachment of various functional groups at different positions, thus serving as a versatile building block for the creation of novel molecules and advanced materials. Notably, the rigid framework of triptycene and its derivatives finds application in materials science, giving rise to materials with remarkable mechanical and electronic properties, thereby contributing to the development of high-performance polymers and organic electronic devices [18,19,20,21]. However, when used as building blocks for creating porous materials, triptycene-based structures often exhibit fixed and ungovernable porous properties, limiting their adaptability for specific applications. Recently, connecting rigid monomers with flexible linkers showed distinguished methane adsorption under high pressure, which held promise as an efficient storage method for NG in motor vehicles. A noteworthy concept introduced by Jeffrey R. Long and colleagues involves the observation of “gate-opening” behavior in flexible metal-organic frameworks (MOFs). This behavior is characterized by distinctive “S-shaped” or “stepped” adsorption isotherms, which have proven advantageous for methane storage applications [22]. Recently, Cafer T. Yavuz et al. constructed a carbon-carbon bonded HCP (COP-150) by benzene and 1,2-dichloroethane as both solvent and flexible linker, achieving the targets the US Department of Energy (DOE) set with high deliverable methane capacity (0.625 g g^−1^ and 294 L L^−1^), which benefited from the flexible mechanism [23]. The flexibility affords the transformation of HCP networks depending on gaseous species from a nonporous or low-porous system to a porous system, which is highlighted for gas adsorption [24,25,26].

**Figure 1:**
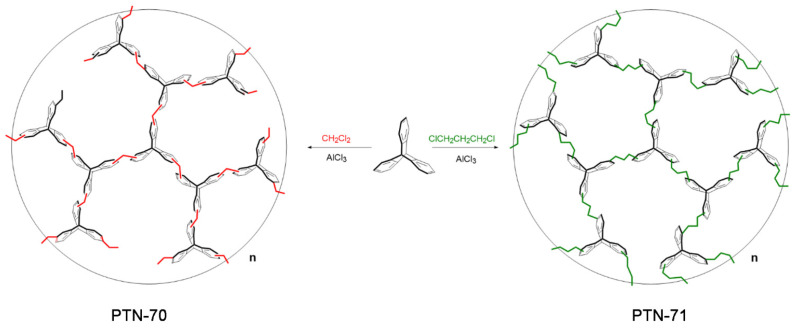
Synthesis of PTN-70 and PTN-71.

The inherent challenge of achieving a delicate balance between maintaining the structural integrity of a network while incorporating highly flexible groups remains a significant hurdle in material science. The total collapse of a network often arises when the structural units predominantly consist of highly flexible groups. To address this challenge, an effective strategy involves a complementary approach that combines a rigid, contorted skeleton with flexible chain segments. This approach has proven to be instrumental in overcoming the structural collapse associated with highly flexible frameworks [27,28,29]. Previous reports from our group highlighted the successful synthesis of diverse hypercrosslinked polymers (HCPs) based on triptycene and its derivatives as monomers, characterized by their three-dimensional rigid paddle wheel shape. These HCPs demonstrated excellent performance in adsorbing organic dyes and exhibited high CO_2_ uptake owing to their superior surface area and porosity [30,31,32,33,34]. Building on this foundation, the present work aimed to explore the distinctiveness of methane adsorption behavior between rigid and flexible linkers in the networks of HCPs (Figure 1). A series of triptycene-based HCPs were synthesized in different solvents, namely dichloromethane and 1,3-dichloropropane, to systematically investigate the impact of linker flexibility on methane adsorption. The synthesis process was designed to be simultaneously convenient and economically feasible, occurring at room temperature without the need for expensive metal catalysts or intricate purification steps. Intriguingly, PTN-71, characterized by a significantly lower surface area (S_BET_ = 574 m^2^ g^−1^) compared to PTN-70 (S_BET_ = 1873 m^2^ g^−1^), exhibited a notably higher methane adsorption capacity (329 m^3^ g^−1^). This unexpected result underscores the crucial role of the flexible mechanism within PTN-71, activated under pressure ranging from 0.05 to 100 bars, in achieving enhanced methane adsorption despite the surface area disparity. The systematic exploration of different solvents and the tailored synthesis approach contribute to unraveling the intricate interplay between structural design, flexibility, and gas adsorption capabilities in hypercross-linked polymers. The outcomes of this study not only advance our understanding of material science but also offer insights into the development of cost-effective and efficient strategies for methane storage, opening avenues for sustainable energy applications.

## 2. Materials and Methods

### 2.1. Materials and Instrumentation

All reagents utilized in this study were procured from commercial suppliers and employed without additional purification. The 13C CP/MAS NMR spectra were acquired with a contact time of 2 ms (ramp 100) and a pulse delay of 3 s.

The X-ray intensity data for PTN-70 and PTN-71 were collected using a standard Bruker SMART-1000 CCD Area Detector System, equipped with a normal-focus Cu-target X-ray tube (Bruker, Germany). Fourier-transform infrared (FT-IR) spectra were recorded on a Bruker model VERTEX 70 infrared spectrometer.

Thermogravimetric analysis (TGA) measurements were conducted on a PerkinElmer model Pyrisl TGA (PerkinElmer Instruments, Shanghai, China) under a nitrogen atmosphere, heating to 800 °C at a rate of 10 °C min^−1^. Surface areas and pore size distributions were determined through nitrogen adsorption and desorption at 77 K, utilizing a Micromeritics ASAP 2020 volumetric adsorption analyzer (PerkinElmer Instruments, Shanghai, China) under a nitrogen atmosphere, heating to 800 °C at a rate of 10 °C min^−1^. Surface areas and pore size distributions were determined through nitrogen adsorption and desorption at 77 K, utilizing a Micromeritics ASAP 2020 volumetric adsorption analyzer (Micromeritics Instrument Crop, Shanghai, China).

The specific rotation of both monomers and polymers was assessed using Autopol IV. Field-emission scanning electron microscopy (FE-SEM) measurements were carried out on a Tescan VEGA 3 SBH field-emission scanning electron microscope (Tescan, Shanghai, China). Transmission electron microscopy (TEM) studies were performed using a Tecnai G220 electron microscope (FEI, America).

### 2.2. Synthesis of PTN-70 and PTN-71

#### 2.2.1. Synthesis of PTN-70

In this synthetic procedure, triptycene (3.18 g, 12.5 mmol, 1 equiv) and AlCl_3_ (6.72 g, 50 mmol, 4 equiv) were meticulously dissolved in 80 mL of dichloromethane within an argon atmosphere. The resultant solution mixture underwent stirring at room temperature for a duration of 3 days. Subsequently, to bring the reaction to completion, 150 mL of methanol was introduced into the mixture. The ensuing suspension was then subjected to filtration, followed by thorough washing with methanol and dichloromethane, each performed three times. To further refine the product, Soxhlet extraction with methanol and dichloromethane was carried out over a period of 3 days. Finally, the resulting product was subjected to vacuum drying for 2 days, ultimately yielding 3.07 g (89%).

#### 2.2.2. Synthesis of PTN-71

In this synthetic procedure, triptycene (3.18 g, 12.5 mmol, 1 equiv) and AlCl_3_ (6.72 g, 50 mmol, 4 equiv) were meticulously dissolved in 80 mL of 1,3-dichloropropane within an argon atmosphere. The resultant solution mixture underwent stirring at room temperature for a duration of 3 days. Subsequently, to bring the reaction to completion, 150 mL of methanol was introduced into the mixture. The ensuing suspension was then subjected to filtration, followed by thorough washing with methanol and dichloromethane, each performed three times. To further refine the product, Soxhlet extraction with methanol and dichloromethane was carried out over a period of 3 days. Finally, the resulting product was subjected to vacuum drying for 2 days, ultimately yielding 2.80 g (66%).

## 3. Results

Triptycene, an exceptional and inherently rigid aromatic compound, served as a pivotal precursor in the intricate synthesis of two distinct hypercrosslinked polymers, designated as PTN-70 and PTN-71. This synthetic endeavor unfolded within the realm of organic chlorinated solvents, with dichloromethane and 1,3-dichloropropane assuming dual roles as both reactants and linkers in the formation of these polymers. The catalytic influence of aluminum chloride emerged as a critical factor, orchestrating the polymerization reactions with finesse, and all of this was orchestrated at room temperature. The nuanced structural characteristics of PTN-70 and PTN-71 demanded a meticulous exploration, prompting a comprehensive array of characterizations. This included the application of sophisticated techniques such as Fourier-transform infrared (FT-IR) spectroscopy and solid-state nuclear magnetic resonance (^13^C CP/MAS NMR) to unravel the intricacies of their molecular architectures. As shown in Figure S1, the FT-IR spectra of PTN-70 and PTN-71, when compared to that of the triptycene, revealed a series of distinctive features. Notably, both polymers exhibited prominent peaks in the range of 600–900 cm^−1^, corresponding to the aromatic C-H bending vibrations, indicative of the presence of aromatic moieties in their structures. Additionally, aromatic C-C stretching peaks were observed at approximately 1500 cm^−1^, further confirming the retention of the aromatic character. The aromatic C-H stretching vibrations, occurring above 3000 cm^−1^, signified the continued existence of aromatic rings within the polymer structures. However, the most conspicuous differences were observed in the regions associated with methylene groups. In both PTN-70 and PTN-71, symmetric and asymmetric C-H stretching bands of methylene were clearly evident at 2856 and 2927 cm^−1^, respectively, indicative of the presence of these methylene units in the polymer backbone. Furthermore, the -CH-bending peaks of methylene were observed at 1380 cm^−1^, further reinforcing the conclusion that methylene groups played a significant role in the polymerization process, likely serving as integral components of the linkers that connected the triptycene units. The spectroscopic results obtained from various techniques collectively provide compelling evidence affirming the successful synthesis of PTN-70 and PTN-71, emphasizing the preservation of the triptycene core and the incorporation of methylene linkers into their respective structures. In the domain of ^13^C CP/MAS NMR spectroscopy (Figure S2), a rich array of signals emerges; particularly notable are the aromatic carbon resonances at 127, 132, and 141 ppm, along with the distinctive signal arising from the methylidyne bridge carbon in triptycene, approximately 80 ppm. The signals observed in the 10–55 ppm range have been confidently assigned to methylene groups, with their broad signal range attributed to varying degrees of shielding effects, resulting in distinct chemical environments for these methylene moieties. Notably, in comparison to PTN-70, the heightened flexibility introduced by 1,3-dichloropropane in PTN-71 facilitates the creation of a higher packing density within the material. Consequently, this enhanced packing density contributes to stronger shielding effects, leading to a shift of the methylene signals in PTN-71 towards the high-field region. Furthermore, an intriguing signal beyond 170 ppm emerges, attributed to the presence of CO_2_ infiltrating the porous networks when exposed to atmospheric air. These comprehensive spectroscopic findings not only shed light on the distinct structural attributes and chemical compositions of the synthesized materials but also underscore the potential for varied applications of PTN-70 and PTN-71 in the realm of porous materials and gas adsorption.

**Figure 2:**
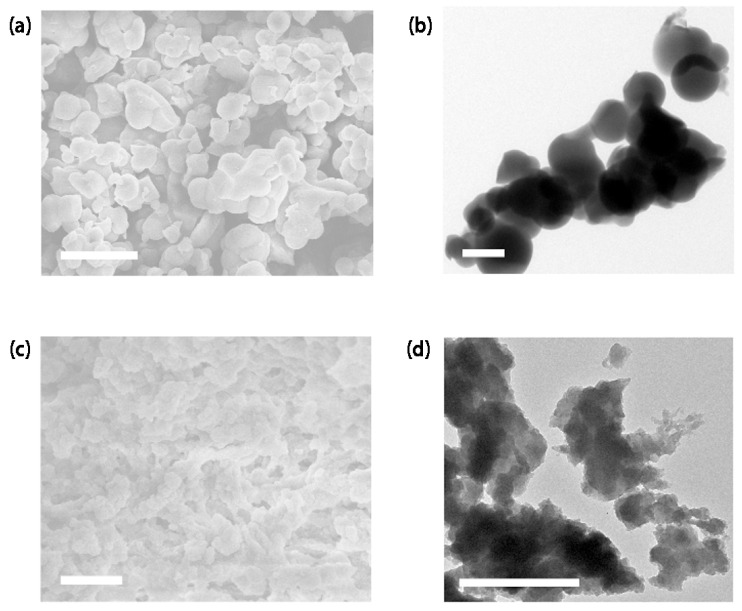
SEM and TEM images of PTN-70 (**a**,**b**) and PTN-71 (**c**,**d**). Scale bar: 5 μm (**a**) and 1 μm (**b**–**d**).

In a dedicated pursuit of unraveling the intricate morphological characteristics inherent in PTN-70 and PTN-71, a sophisticated approach was adopted, leveraging advanced imaging techniques, specifically field-emission scanning electron microscopy (FE-SEM) and transmission electron microscopy (TEM). The outcome of these meticulous analyses provided a wealth of insights into the structural attributes of these hypercrosslinked polymers, unveiling visually intriguing features that characterize both PTN-70 and PTN-71. The captivating images presented in Figure 2 and Figure S3 offered a glimpse into the world of amorphous, rough spherical particles, each adorned with irregular structures. What emerges as particularly striking in this exploration is the marked distinction observed in the morphology between PTN-70 and PTN-71. PTN-71, in particular, commands attention due to the notably higher packing density of its amorphous, rough spherical particles in comparison to its counterpart, PTN-70. This dissimilarity in packing density, evident in the vivid imagery captured by the advanced microscopy techniques, was further substantiated by the outcomes of powder X-ray diffraction (PXRD) analyses, as eloquently showcased in Figure S4. The PXRD analyses laid bare broad peaks, a characteristic feature that unequivocally signifies the noncrystalline nature inherent in both PTN-70 and PTN-71.

The exploration of the thermal stability of PTN-70 and PTN-71, conducted through thermogravimetric analysis (TGA), served as a pivotal dimension in the characterization of these materials. The TGA results, presented in the insightful Figure S5, unveiled a notable stability in both polymers, with PTN-70 exhibiting resilience up to temperatures of 400 °C and PTN-71 up to 350 °C. However, the intriguing observation during the TGA analysis surfaced at 150 °C, where a marginal decrease in mass was noted. This phenomenon was attributed to the persistence of solvent trapped within the micropore structure of the polymers. The solvent, proving recalcitrant to removal under vacuum conditions at room temperature, presented a significant challenge in achieving the desired purity of the materials. A subsequent treatment at 100 °C in a vacuum for 10 h proved to be an effective remedy, facilitating the complete removal of the persistent solvent within the micropore structure.

**Figure 3:**
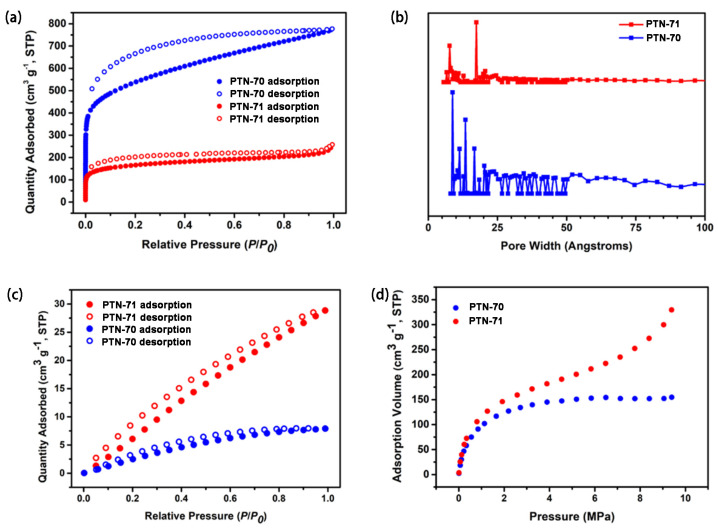
Nitrogen sorption isotherms of PTN-70 and PTN-71 at 77 K (**a**), pore size distributions calculated using NLDFT methods (**b**). (**c**) Methane (273 K) isotherm of PTN-70 and PTN-71. (**d**) Methane adsorption of PTN-70 and PTN-71 up to 95 bars at 273 K.

The meticulous examination of the pore properties of hypercrosslinked polymers (HCPs) unfolded with a thorough process involving desolvation at 120 °C for an extensive 10-h duration under vacuum, followed by nitrogen sorption and desorption analysis at a temperature of 77 K, as thoughtfully illustrated in Figure 3a. A critical aspect of this assessment was the scrutiny of the Brunauer–Emmett–Teller (BET) surface area, a pivotal parameter elucidating the accessibility of the internal surface of the polymer to nitrogen gas. The results portrayed a compelling narrative of contrast between PTN-70 and PTN-71, with the former exhibiting a notably larger surface area, measuring an impressive 1873 m^2^ g^−1^. In stark contrast, PTN-71 registered at 574 m^2^ g^−1^, indicating a considerable reduction in surface area compared to its counterpart. The Langmuir surface area, offering insights into monolayer coverage, accentuated this difference, with PTN-70 showcasing a Langmuir surface area of 2525 m^2^ g^−1^, surpassing the 770 m^2^ g^−1^ observed for PTN-71 (Figures S6 and S7). Moreover, the exploration extended to the determination of the total pore volume, a pivotal metric influencing the storage capacity of gases within the material. The discernment that PTN-70 displayed a notably higher total pore volume, measuring 1.20 cm^3^ g^−1^, compared to the 0.40 cm^3^ g^−1^ observed for PTN-71, indicated that PTN-70 harbors a more extensive network of internal voids, potentially offering enhanced storage capacity for gases.

A pivotal consideration in this nuanced evaluation was the integration of a flexible linker within PTN-71, specifically 1,3-dichloropropane. This flexible linker has the potential to shape the creation of denser structures within the polymer, potentially influencing the diffusion of nitrogen gas within the material. This observation resonated harmoniously with the findings from morphological characterization, providing a plausible explanation for the reduction in both surface area and pore volume observed in PTN-71 compared to PTN-70. The nitrogen adsorption isotherms delved further into the intricacies of the pore structures of PTN-70 and PTN-71. A discernible surge in nitrogen gas uptake at low relative pressure (P/P_0_ < 0.001) underscored the presence of abundant micropore structures within the HCPs. This phenomenon reflects the materials’ adeptness at effectively adsorbing gas molecules within their intricate networks of sub-nanometer-sized pores. The observed hysteresis with higher capacity hinted at the irreversible uptake of gas molecules at mesopores or through pore entrances, highlighting the swelling behavior intrinsic to the polymer skeleton—a recurrent trait in amorphous microporous polymer networks. Such insights accentuate the dynamic nature of these materials and their capacity to undergo structural changes in response to gas adsorption.

The investigation was further enriched through nonlocal density functional theory (NLDET) utilizing the Micromeritics ASAP 2020 volumetric adsorption analyzer. This analytical approach served to corroborate the prevalence of micropores as the predominant structural component within the HCPs, as visually exemplified in Figure 3b. The analysis provided a comprehensive visualization of the pore size distributions of PTN-70 and PTN-71, predominantly falling within the microporous range, with dimensions consistently below 20 Å. This unequivocally solidified their classification as microporous polymers, boasting intricate networks of sub-nanometer-sized pores. However, a pivotal distinction surfaced between PTN-70 and PTN-71 in terms of mesoporous characteristics. PTN-70 exhibited the presence of mesopores within the range of 2–50 nm, contributing to a more diverse pore structure. In contrast, PTN-71 did not manifest such mesoporous features. This divergence can be attributed to the influential role played by the flexible linker, 1,3-dichloropropane. The flexible nature of this linker facilitated a more compact packing of polymer chains within PTN-71, as evidenced by the FE-SEM and TEM analyses, resulting in a discernible decrease in mesoporous features. This nuanced insight underscores the intricate interplay between the choice of linker and the resulting pore characteristics in hypercrosslinked polymers, shedding light on the material’s ability to tailor its structural attributes based on the chemical composition.

In the assessment of natural gas (NG) adsorption performance for the two distinct polymers, PTN-70 and PTN-71, rigorous experiments were conducted by subjecting them individually to pure methane gas at a controlled temperature of 273 K. As shown in Figure 3c, despite the fact that the BET surface area of PTN-71 is obviously inferior to that of PTN-70, the methane adsorption capacity is much better, reaching 28.8 cm^3^ g^−1^ at 273 K/bar, while the methane adsorption capacity of PTN-70 is 7.9 cm^3^ g^−1^ at 273 K/bar. A pronounced hysteresis effect was observed in atmospheric methane adsorption and desorption measurements, corroborating the role of the flexible network structure in this distinct adsorption behavior. The methane adsorption isotherms unveiled intriguing and disparate behaviors. Upon increasing the pressure to 95 bars, PTN-70 exhibited a rapid and substantial increase in methane adsorption, swiftly ascending from 0 to 20 bars, whereafter the adsorption capacity reached a plateau, remaining relatively constant despite the continuous elevation of gas pressure (Figure 3d). In stark contrast, the methane adsorption isotherm for PTN-71 displayed a distinctive two-step pattern. After an initial phase, methane uptake experienced a sharp resurgence when the pressure reached 45 bars, intriguingly continuing to rise even at high pressures and ultimately saturating at 95 bars. This intriguing behavior was attributed to the swelling response of the flexible network structure within PTN-71 when subjected to elevated pressure conditions. Cycling tests were performed to assess the methane adsorption capabilities of two different polymers, PTN-70 and PTN-71, revealing their consistent and stable methane adsorption capacities (Figure S8). Importantly, this behavior provided a plausible explanation for why PTN-71, despite having a significantly lower surface area, exhibited a notably higher methane adsorption capacity under high gas pressure compared to PTN-70 [15,21].

**Figure 4:**
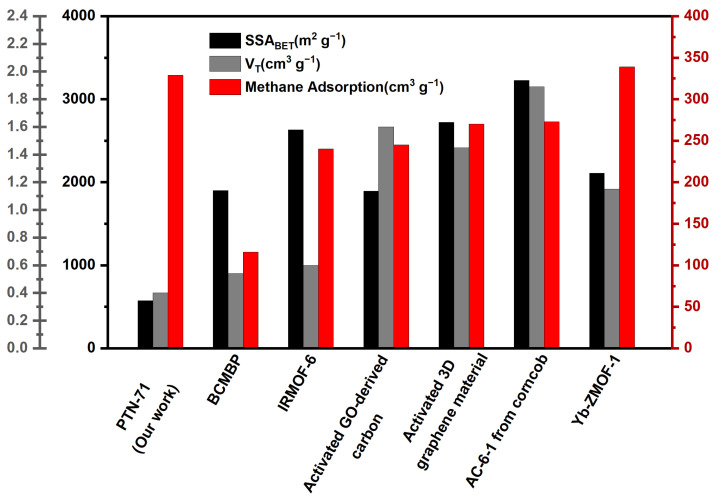
Adsorption capacity of methane for various materials recently reported. Details are located in Table S1 of the Supplementary Materials.

An exploration into the properties of PTN-70 and PTN-71 has yielded a significant and intriguing revelation—their shared ability for reversible methane storage. This finding carries profound implications within the domain of adsorbed natural gas (ANG) technology, challenging longstanding assumptions in the field. Traditionally, the prevailing belief asserted a direct correlation between methane adsorption capacity and specific surface area, as well as total pore volume. Higher values of these parameters were generally expected to result in superior gas storage performance, as illustrated in Figure 4. However, PTN-71 has defied this conventional wisdom, emerging as a compelling exception to the established trend. Contrary to expectations, it exhibited remarkable reversible methane storage capabilities despite not conforming to the anticipated correlation between methane adsorption capacity, specific surface area, and total pore volume. This unexpected behavior prompts a reevaluation of existing paradigms in the field of ANG technology, challenging the notion that higher specific surface area and total pore volume invariably lead to enhanced gas storage performance [35,36,37,38,39,40].

An illuminating point of comparison arises when juxtaposing the findings with the recent material Yb-ZMOF-1, celebrated for its exceptionally specific surface area, surpassing 1000 m^2^ g^−1^ [40]. Intriguingly, PTN-71 has exhibited a methane adsorption capacity comparable to that of Yb-ZMOF-1, despite possessing a notably lower specific surface area and pore volume. This revelation serves as a striking testament to the influential role played by the flexible network structure within PTN-71. The surprising parity in methane adsorption between PTN-71 and Yb-ZMOF-1, despite substantial differences in structural characteristics, underscores the pivotal importance of the inherent flexibility within PTN-71. This flexibility appears to be a key factor contributing to its efficiency as a methane adsorbent, particularly under conditions of elevated gas pressure. The traditional paradigm linking superior gas storage performance to higher specific surface area and pore volume is thus challenged by the exceptional behavior exhibited by PTN-71.

The unprecedented methane storage capabilities demonstrated by PTN-71 challenge conventional assumptions within the field, offering a fresh perspective that accentuates the crucial role played by material flexibility and structure in methane adsorption. This insight opens novel avenues for the design of highly effective gas storage materials, suggesting that flexibility may be a crucial factor in optimizing performance under varying conditions. This groundbreaking discovery not only advances our understanding of methane adsorption mechanisms but also holds promise for the development of more efficient, flexible, and adaptable materials for adsorbed natural gas (ANG) technology [30].

## 4. Conclusions

In summary, our study harnessed the unique properties of triptycene as rigid building blocks to judiciously craft two distinct physicochemically stable triptycene-based hypercrosslinked polymers, denoted as PTN-70 and PTN-71, employing a cost-effective and expedient synthesis process under mild conditions. A comparative analysis between PTN-70, characterized by a rigid methylene linker, and PTN-71, featuring a flexible propylene linker, revealed intriguing findings. Despite PTN-71 exhibiting a lower BET surface area in contrast to the rigidly linked PTN-70, it demonstrated a remarkable and unexpected superiority in methane adsorption performance. The methane adsorption capacity of PTN-71 significantly outperformed that of PTN-70, reaching an impressive 329 cm³ g⁻¹, particularly under high-pressure conditions. This exceptional performance can be attributed to the unique flexible network structure of PTN-71, which undergoes a swelling response when subjected to elevated pressure conditions, facilitating enhanced methane adsorption. The flexibility introduced by the propylene linker played a pivotal role in expanding the porous network of PTN-71, presenting a stark contrast to the rigid network of PTN-70. This breakthrough challenges conventional assumptions by highlighting the potential of flexible-linked hypercrosslinked polymers (HCPs) as highly efficient methane adsorbents. The study not only expands the understanding of gas storage materials but also opens new avenues for advanced methane storage technologies with substantial implications for clean energy and environmental sustainability. The remarkable methane adsorption performance of PTN-71 underscores the versatility and adaptability of hypercrosslinked polymers, offering a promising avenue for the development of efficient and environmentally friendly methane storage solutions that can contribute significantly to the transition towards cleaner energy sources and sustainable practices.

## Data Availability

Research data are available in this document and in the related Supplementary Materials submitted.

## Supplementary Materials

The following supporting information can be downloaded at: https://www.mdpi.com/article/10.3390/polym16010156/s1, Figure S1: FT-IR spectroscopy of triptycene, PTN-70 and PTN-71; Figure S2: ^13^C MAS NMR spectra of (a) PTN-70 (blue) and (b) PTN-71 (red); Figure S3: SEM and TEM images of PTN-70 (a and b) and PTN-71 (c and d). Scale bar: 0.2 μm; Figure S4: Powder X-ray diffraction spectra of (a) PTN-70 and (b) PTN-71; Figure S5: Thermogravimetric analysis of PTN-70 and PTN-71 at room temperature (a) and after been treated at 100 ℃ in the vacuum for 10 h (b); Figure S6: The Brunauer-Emmett-Teller (BET) surface areas of PTN-70 calculated from nitrogen sorption analysis at 77 K; Figure S7: The Brunauer-Emmett-Teller (BET) surface areas of PTN-70 calculated from nitrogen sorption analysis at 77 K; Figure S8: The cycling test of methane adsorption of PTN-70 and PTN-71 up to 95 bars at 273 K; Table S1: Adsorption capacity of methane for various materials recently reported. References [35,36,37,38,39,40] are cited in the Supplementary Materials.

## References

[1] Menon V.C., Komarneni S. (1998). Porous Adsorbents for Vehicular Natural Gas Storage: A Review. J. Porous Mater..

[2] Zou X.Y., Xue R., An Z.W., Li H.W., Zhang J.L., Jiang Y., Huang L.J., Wu W., Wang S.F., Hu G.-H. (2023). Recent Advances in Flexible CNC-Based Chiral Nematic Film Materials. Small.

[3] Xue R., Zhao H., An Z.W., Wu W., Jiang Y., Li P., Huang C.-X., Shi D., Li R.K.Y., Hu G.-H. (2023). Self-healable, solvent response cellulose nanocrystal/ waterborne polyurethane nanocomposites with encryption capability. ACS Nano.

[4] Connolly B.M., Madden D., Wheatley A., Fairen-Jimenez D. (2020). Shaping the Future of Fuel: Monolithic Metal-Organic Frameworks for High-Density Gas Storage. J. Am. Chem. Soc..

[5] Rowland C.A., Lorzing G.R., Gosselin E.J., Trump B.A., Yap G.P.A., Brown C.M., Bloch E.D. (2018). Methane Storage in Paddlewheel-Based Porous Coordination Cages. J. Am. Chem. Soc..

[6] Acharyya K., Mukherjee P.S. (2019). Organic Imine Cages: Molecular Marriage and Applications. Angew. Chem. Int. Ed..

[7] Bracco S., Piga D., Bassanetti I., Perego J., Comotti A., Sozzani P. (2017). Porous 3D polymers for high pressure methane storage and carbon dioxide capture. J. Mater. Chem. A.

[8] Che S., Pang J.D., Kalin A.J., Wang C.X., Ji X.Z., Lee J., Cole D., Li J.L., Tu X.M., Zhang Q. (2020). Rigid Ladder-Type Porous Polymer Networks for Entropically Favorable Gas Adsorption. ACS Mater. Lett..

[9] Zhang A.J., Zhang Q.K., Bai H., Li L., Li J. (2014). Polymeric nanoporous materials fabricated with supercritical CO_2_ and CO_2_-expanded liquids. Chem. Soc. Rev..

[10] Zhu T.T., Pei B.Y., Di T., Xia Y.X., Li T.S., Li L. (2020). Thirty-minute preparation of microporous polyimides with large surface areas for ammonia adsorption. Green Chem..

[11] Makal T.A., Li J.R., Lu W.G., Zhou H.C. (2012). Methane storage in advanced porous materials. Chem. Soc. Rev..

[12] Liu Y.Z., Li S., Dai L., Li J.N., Lv J.N., Zhu Z.J.J., Yin A.X., Li P.F., Wang B. (2021). The Synthesis of Hexaazatrinaphthylene-Based 2D Conjugated Copper Metal-Organic Framework for Highly Selective and Stable Electroreduction of CO_2_ to Methane. Angew. Chem. Int. Ed..

[13] Kumar K.V., Preuss K., Titirici M.M., Rodriguez-Reinoso F. (2017). Nanoporous Materials for the Onboard Storage of Natural Gas. Chem. Rev..

[14] Fang H., Zheng B., Zhang Z.H., Li H.X., Xue D.X., Bai J.F. (2021). Ligand-Conformer-Induced Formation of Zirconium-Organic Framework for Methane Storage and MTO Product Separation. Angew. Chem. Int. Ed..

[15] Zhang L., Sun T., Dong Y.B., Fang Z.B., Xu Y.X. (2022). Electron-donating group induced rapid synthesis of hyper-crosslinked polymers. Sci. Bull..

[16] Tan L.X., Tan B. (2017). Hypercrosslinked porous polymer materials: Design, synthesis, and applications. Chem. Soc. Rev..

[17] Lee J.S.M., Briggs M.E., Hu C.C., Cooper A.I. (2018). Controlling electric double-layer capacitance and pseudocapacitance in heteroatom-doped carbons derived from hypercrosslinked microporous polymers. Nano Energy.

[18] Liu N., Ma H., Sun R., Zhang Q.-P., Tan B., Zhang C. (2023). Porous Triptycene Network Based on Tröger’s Base for CO_2_ Capture and Iodine Enrichment. ACS Appl. Mater. Interfaces.

[19] Eckstein B.J., Brown L.C., Noll B.C., Moghadasnia M.P., Balaich G.J., McGuirk C.M. (2021). A Porous Chalcogen-Bonded Organic Framework. J. Am. Chem. Soc..

[20] Soto C., Torres-Cuevas E.S., Gonzalez-Ortega A., Palacio L., Pradanos P., Freeman B.D., Lozano A.E., Hernandez A. (2021). Hydrogen Recovery by Mixed Matrix Membranes Made from 6FCl-APAF HPA with Different Contents of a Porous Polymer Network and Their Thermal Rearrangement. Polymers.

[21] Zotkin M.A., Alentiev D.A., Shorunov S.V., Sokolov S.E., Gavrilova N.N., Bermeshev M.V. (2023). Microporous polynorbornenes bearing carbocyclic substituents: Structure-property study. Polymer.

[22] Mason J.A., Oktawiec J., Taylor M.K., Hudson M.R., Rodriguez J., Bachman J.E., Gonzalez M.I., Cervellino A., Guagliardi A., Brown C.M. (2015). Methane storage in flexible metal-organic frameworks with intrinsic thermal management. Nature.

[23] Rozyyev V., Thirion D., Ullah R., Lee J., Jung M., Oh H., Atilhan M., Yavuz C.T. (2019). High-capacity methane storage in flexible alkane-linked porous aromatic network polymers. Nat. Energy.

[24] Llewellyn P.L., Bourrelly S., Serre C., Filinchuk Y., Férey G. (2006). How Hydration Drastically Improves Adsorption Selectivity for CO_2_ over CH_4_ in the Flexible Chromium Terephthalate MIL-53. Angew. Chem. Int. Ed..

[25] Horike S., Shimomura S., Kitagawa S. (2009). Soft porous crystals. Nat. Chem..

[26] Zhan Z., Yu J.C., Li S.Q., Yi X.X., Wang J.Y., Wang S.L., Tan B. (2023). Ultrathin Hollow Co/N/C Spheres from Hyper-Crosslinked Polymers by a New Universal Strategy with Boosted ORR Efficiency. Small.

[27] Qiao S., Li Z., Zhang B., Li Q., Jin W., Zhang Y., Wang W., Li Q., Liu X. (2019). Flexible chain & rigid skeleton complementation polycarbazole microporous system for gas storage. Micropor. Mesopor. Mater..

[28] An Z.-W., Ye K., Xue R., Zhao H., Liu Y., Li P., Chen Z.-M., Huang C.-X., Hu G.-H. (2023). Recent advances in self-healing polyurethane based on dynamic covalent bonds combined with other self-healing methods. Nanosacle.

[29] Li H.-W., Zhang J.-L., Xue R., An Z.-W., Wu W., Liu Y., Hu G.-H., Zhao H. (2023). Construction of self-healable and recyclable waterborne polyurethane-MOF membranes for adsorption of dye wastewater. Sep. Purif. Technol..

[30] Dai L., Dong A.W., Meng X.J., Liu H.Y., Li Y.T., Li P.F., Wang B. (2023). Enhancement of Visible-Light-Driven Hydrogen Evolution Activity of 2D pi-Conjugated Bipyridine-Based Covalent Organic Frameworks via Post-Protonation. Angew. Chem. Int. Ed..

[31] Li M.P., Ren H., Sun F.X., Tian Y.Y., Zhu Y.L., Li J.L., Mu X., Xu J., Deng F., Zhu G.S. (2018). Construction of Porous Aromatic Frameworks with Exceptional Porosity via Building Unit Engineering. Adv. Mater..

[32] Zu Y.C., Li J.W., Li X.L., Zhao T.Y., Ren H., Sun F.X. (2022). Imine-linked porous aromatic frameworks based on spirobifluorene building blocks for CO_2_ separation. Micropor. Mesopor. Mater..

[33] Zhang Q.P., Wang Z., Zhang Z.W., Zhai T.L., Chen J.J., Ma H., Tan B., Zhang C. (2021). Triptycene-based Chiral Porous Polyimides for Enantioselective Membrane Separation. Angew. Chem. Int. Ed..

[34] Wang Z., Ma H., Zhai T.L., Cheng G., Xu Q., Liu J.M., Yang J.K., Zhang Q.M., Zhang Q.P., Zheng Y.S. (2018). Networked Cages for Enhanced CO_2_ Capture and Sensing. Adv. Sci..

[35] Wood C.D., Tan B., Trewin A., Su F., Rosseinsky M.J., Bradshaw D., Sun Y., Zhou L., Cooper A.I. (2008). Microporous Organic Polymers for Methane Storage. Adv. Mater..

[36] Eddaoudi M., Kim J., Rosi N., Vodak D., Wachter J., O’Keeffe M., Yaghi O.M. (2002). Systematic Design of Pore Size and Functionality in Isoreticular MOFs and Their Application in Methane Storage. Science.

[37] Srinivas G., Burress J., Yildirim T. (2012). Graphene oxide derived carbons (GODCs): Synthesis and gas adsorption properties. Energy Environ. Sci..

[38] Mahmoudian L., Rashidi A., Dehghani H., Rahighi R. (2016). Single-step scalable synthesis of three-dimensional highly porous graphene with favorable methane adsorption. Chem. Eng. J..

[39] Liu B.S., Wang W.S., Wang N., Au C.T. (2014). Preparation of activated carbon with high surface area for high-capacity methane storage. J. Energy Chem..

[40] Li H.X., Zhang Z.H., Fang H., Xue D.X., Bai J.F. (2022). Synthesis, structure and high methane storage of pure D6R Yb(Y) nonanuclear cluster-based zeolite-like metal-organic frameworks. J. Mater. Chem. A.

