# Supplementary material for: Rigidity with Flexibility: Porous Triptycene Networks for Enhancing Methane Storage
Source: Polymers (Basel). 2024 Jan 4;16(1):156. doi: 10.3390/polym16010156 (PMC10780442; doi:10.3390/polym16010156)
Supplement: Supplementary file 1 [file polymers-16-00156-s001.zip › polymers-2663525-supplementary.pdf]

Supporting information

Rigidity with Flexibility: Porous Triptycene Networks for Enhancing Methane Storage

Fei Guo [1,†], Hui Ma [2,†], Bin-Bin Yang [2,†], Zhen Wang [1,2,*], Xiang-Gao Meng [3,*], Jian-Hua Bu [4] and Chun Zhang [2]

[1] National Engineering Laboratory for Advanced Yarn and Fabric Formation and Clean Production, Technology Institute, Wuhan Textile University, Wuhan 430200, China; guofeifei0806@163.com

[2] College of Life Science and Technology, National Engineering Research Center for Nanomedicine, Huazhong University of Science and Technology, Wuhan 430074, China; dongji0828@126.com (H.M.); 17371266359@163.com (B.-B.Y.); chunzhang@hust.edu.cn (C.Z.)

[3] School of Chemistry, Central China Normal University, Wuhan 430079, China

[4] Xi'an Modern Chemistry Research Institute, Xi'an 710065, China; bujianhua@163.com

* Correspondence: wz@wtu.edu.cn (Z.W.); xianggao_meng@126.com (X.-G.M.)

† These authors contributed equally to this work.

1. Materials

All reagents were purchased from commercial suppliers and used without further purification.

2. Characterization

The ^{13}C CP/MAS NMR spectra were recorded with a contact time of 2 ms (ramp 100) and pulse delay of 3 s. The X-ray intensity data for PTN-70 and PTN-71 were collected on a standard Bruker SMART-1000 CCD Area Detector System equipped with a normal-focus Cu-target X-ray tube. Fourier transform infrared (FT-IR) spectra were recorded on a Bruker model VERTEX 70 infrared spectrometer. Thermogravimetric Analysis (TGA) measurements were performed on a PerkinElmer model Pyrisl TGA under N_2, by heating to 800 °C at a rate of 10 °C min^{-1}. Surface areas and pore size distributions were measured by nitrogen adsorption and desorption at 77 K using a Micromeritics ASAP 2020 volumetric adsorption analyzer. The specific rotation of monomer and polymer were tested by Autopol IV. Field-emission scanning electron microscopy (FE-SEM) measurements were performed on Tescan VEGA 3 SBH field-emission scanning electron microscope. TEM studies were conducted on a Tecnai G220 electron microscope.

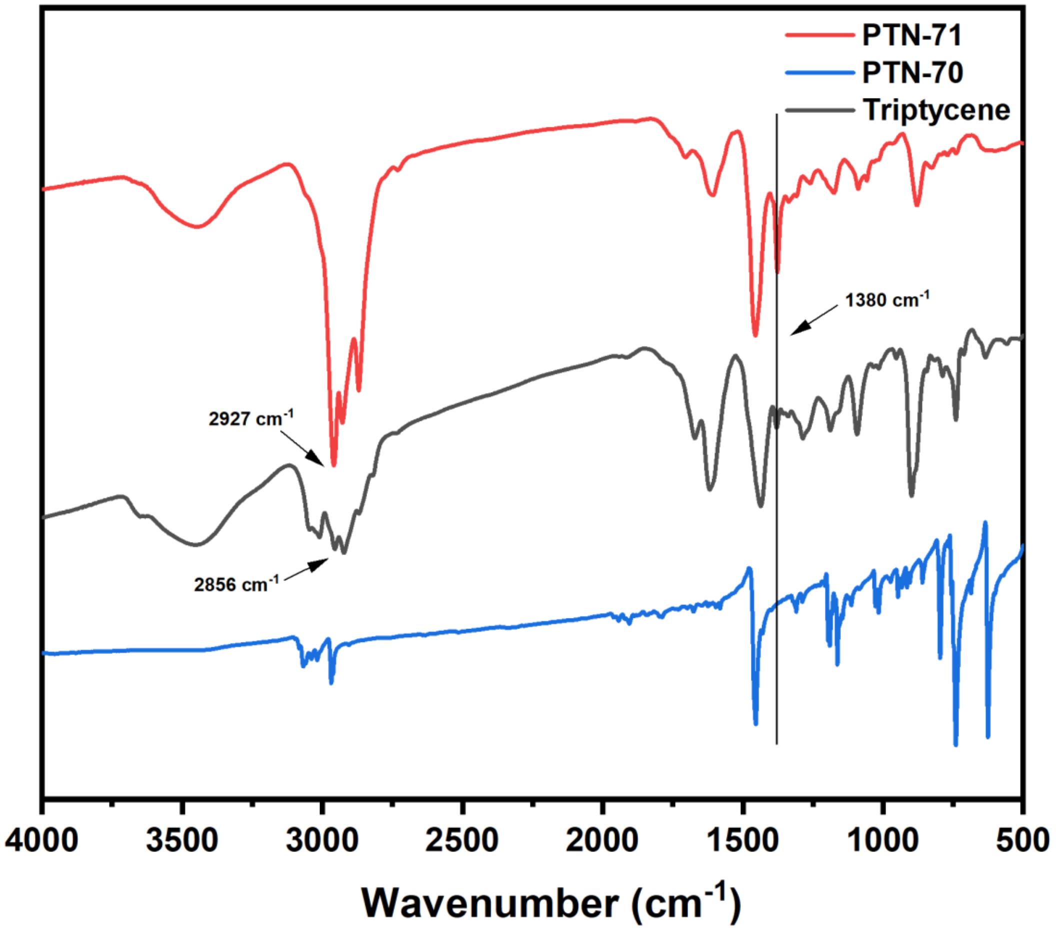

Figure S1. FT-IR spectroscopy of triptycene, PTN-70 and PTN-71.

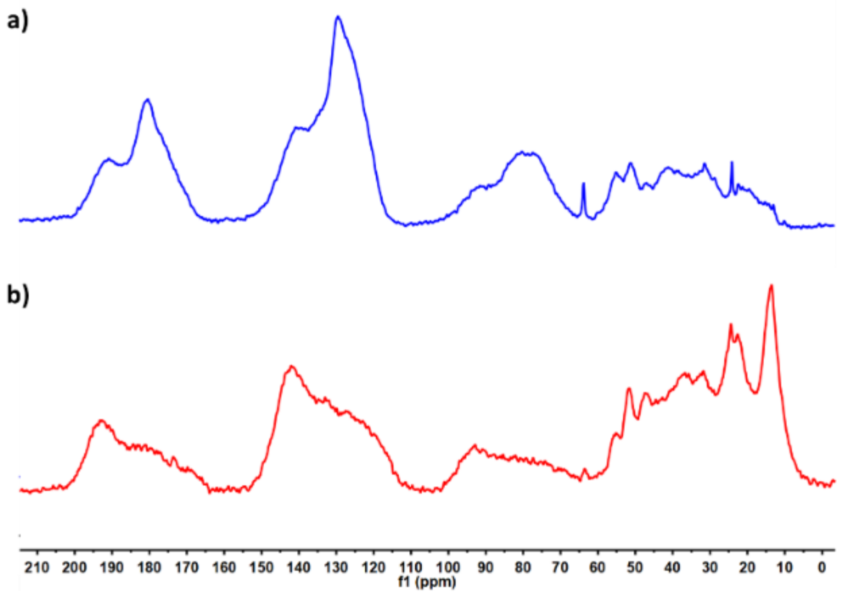

Figure S2. ^{13}C MAS NMR spectra of (a) PTN-70 (blue) and (b) PTN-71(red).

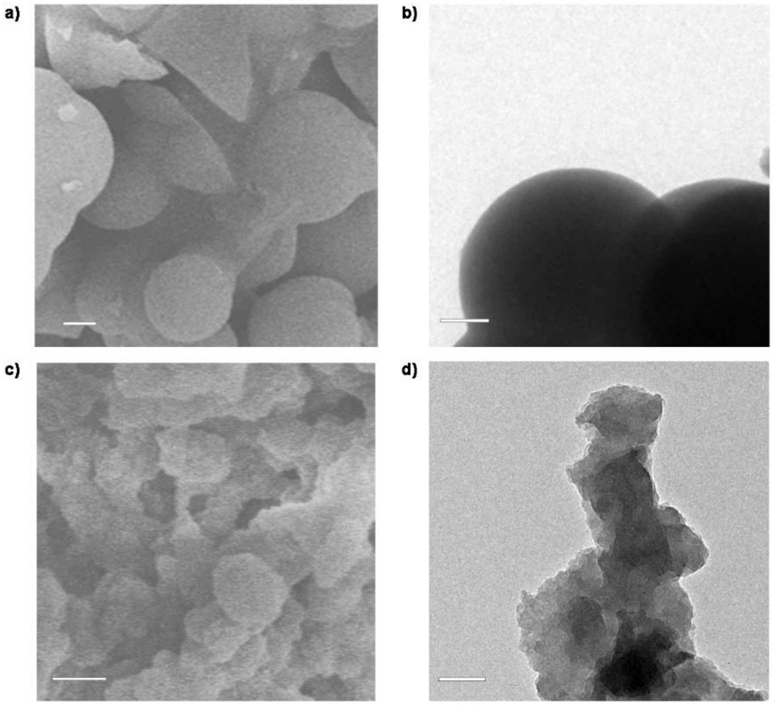

Figure S3. SEM and TEM images of PTN-70 (a and b) and PTN-71 (c and d). Scale bar: 0.2 μm.

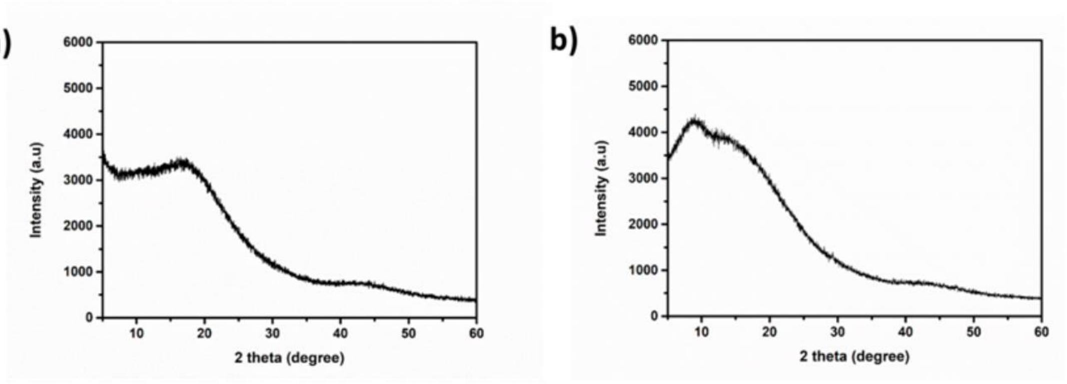

Figure S4. Powder X-ray diffraction spectra of (a) PTN-70 and (b) PTN-71.

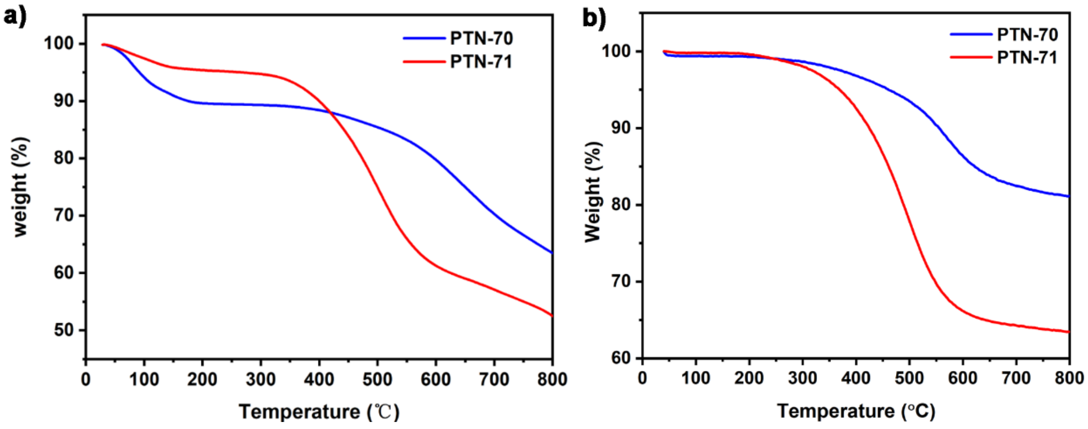

Figure S5. Thermogravimetric analysis of PTN-70 and PTN-71 at room temperature (a) and after been treated at 100 ℃ in the vacuum for 10 h (b).

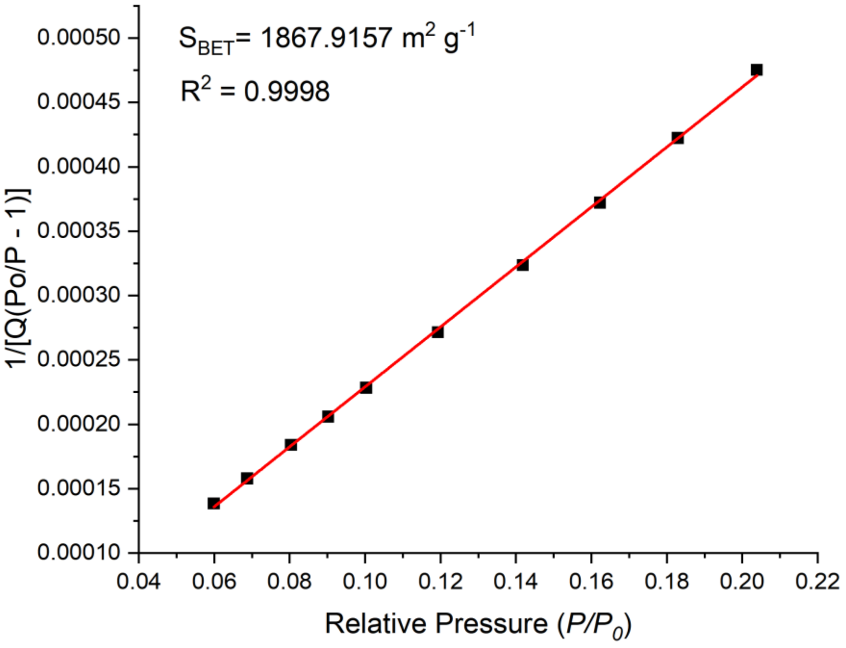

Figure S6. The Brunauer-Emmett-Teller (BET) surface areas of PTN-70 calculated from nitrogen sorption analysis at 77K.

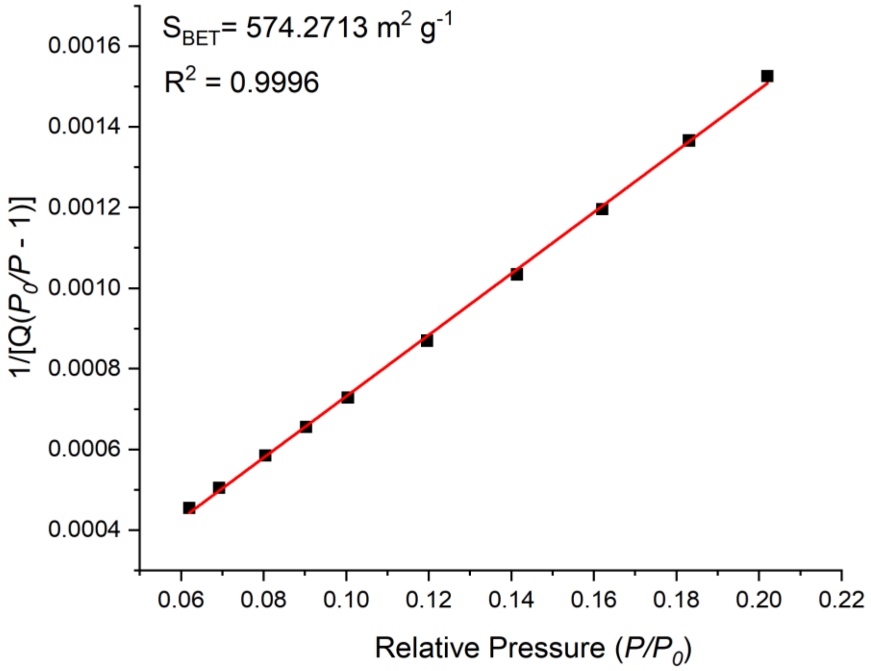

Figure S7. The Brunauer-Emmett-Teller (BET) surface areas of PTN-70 calculated from nitrogen sorption analysis at 77K.

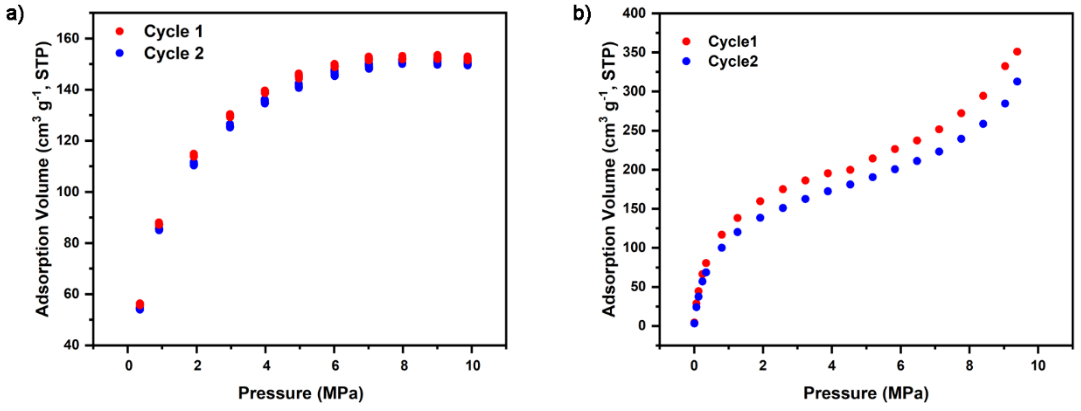

Figure S8. The cycling test of methane adsorption of PTN-70 and PTN-71 up to 95 bars at 273 K.

Table S1. Adsorption capacity of methane for various materials recently reported.

Adsorbent	SSA_{BET} (m^2 g^{-1})	V_T (cm^3 g^{-1})	Methane Adsorption (cm^3 g^{-1})	Ref.
Our work	574	0.40	329	/
BCMBP polymer	1900	0.54	116	[20]
IRMOF-6	2630	0.60	240	[21]
Activated GO-derived carbon	1894	1.60	245	[22]
Activated 3D graphene material	2720	1.45	270	[23]
AC-6-1 from corncob	3227	1.89	273	[24]
Yb-ZMOF-1	2107	1.15	339	[25]

References

(1) Wood, C. D.; Tan, B.; Trewin, A.; Su, F.; Rosseinsky, M. J.; Bradshaw, D.; Sun, Y.; Zhou, L.; Cooper, A. I. Microporous Organic Polymers for Methane Storage. *Adv. Mater.* **2008**, *20* (10), 1916-1921. DOI: https://doi.org/10.1002/adma.200702397.

(2) Eddaoudi, M.; Kim, J.; Rosi, N.; Vodak, D.; Wachter, J.; O'Keeffe, M.; Yaghi, O. M. Systematic Design of Pore Size and Functionality in Isoreticular MOFs and Their Application in Methane Storage. *Science* **2002**, *295* (5554), 469-472. DOI: doi:10.1126/science.1067208.

(3) Srinivas, G.; Burress, J.; Yildirim, T. Graphene oxide derived carbons (GODCs): synthesis and gas adsorption properties. *Energ. Environ. Sci.* **2012**, *5* (4), 6453-6459. DOI: 10.1039/c2ee21100a.

(4) Mahmoudian, L.; Rashidi, A.; Dehghani, H.; Rahighi, R. Single-step scalable synthesis of three-dimensional highly porous graphene with favorable methane adsorption. *Chem. Eng. J.* **2016**, *304*, 784-792. DOI: https://doi.org/10.1016/j.cej.2016.07.015.

(5) Liu, B. S.; Wang, W. S.; Wang, N.; Au, C. T. Preparation of activated carbon with high surface area for high-capacity methane storage. *J. Energy Chem.* **2014**, *23* (5), 662-668. DOI: 10.1016/s2095-4956(14)60198-4.

(6) Li, H. X.; Zhang, Z. H.; Fang, H.; Xue, D. X.; Bai, J. F. Synthesis, structure and high methane storage of pure D6R Yb(Y) nonanuclear cluster-based zeolite-like metal-organic frameworks. *J. Mater. Chem. A* **2022**, *10* (28), 14795-14798. DOI: 10.1039/d2ta02948k.
